# Smoking Behavior and Use of Tobacco Industry Sponsored Websites Among Medical Students and Young Physicians in Argentina

**DOI:** 10.2196/jmir.2528

**Published:** 2014-02-06

**Authors:** M Victoria Salgado, Raul Mejia, Celia P Kaplan, Eliseo J Perez-Stable

**Affiliations:** ^1^Universidad de Buenos AiresBuenos AiresArgentina; ^2^Centro de Estudios de Estado y SociedadBuenos AiresArgentina; ^3^University of California, San FranciscoSan Francisco, CAUnited States

**Keywords:** smoking, medical students, tobacco websites, Latin America

## Abstract

**Background:**

Internet-based marketing has become an attractive option for promoting tobacco products due to its potential to avoid advertising restrictions. In Argentina, several cigarette brands have designed websites for the local market, which promote user participation.

**Objective:**

The intent of the study was to report on the use of tobacco company-sponsored websites by medical students and recently graduated physicians.

**Methods:**

An online self-administered survey was conducted among eligible medical students and recent graduates from the University of Buenos Aires (UBA). Sampling was from lists of email addresses of students enrolled in two required courses. Eligibility criteria were ages 18-30 years and reporting on smoking status. Questions on Internet use included accessing a tobacco brand website at least once during their lifetime and any use of tobacco promotional materials.

**Results:**

The response rate was 35.08% (1743/4969). The final sample included 1659 participants: 73.06% (1212/1659) were women and mean age was 26.6 years (SD 1.9). The majority were current medical students (55.70%, 924/1659) and 27.31% (453/1659) were current smokers. Men were more likely to report having seen a tobacco advertisement on the Internet (*P*=.001), to have received a tobacco promotion personally addressed to them (*P*=.03), to have used that promotion (*P*=.02), and to have accessed a tobacco-sponsored website (*P*=.01). Among respondents, 19.35% (321/1659) reported having accessed a tobacco-sponsored website at least once in their lifetime and almost all of them (93.8%, 301/321) accessed these sites only when it was necessary for participating in a marketing promotion. Most people logging on for promotions reported entering once a month or less (58.9%, 189/321), while 25.5% (82/321) reported accessing the tobacco industry Internet sites once a week or more. In adjusted logistic regression models, participants were more likely to have accessed a tobacco brand website if they were former smokers (OR 2.45, 95% CI 1.42-4.22) or current (OR 8.12, 95% CI 4.66-14.16), if they reported having seen a tobacco advertisement on the Internet (OR 2.44, 95% CI 1.77-3.37), received a tobacco promotion personally addressed to them (OR 5.62; 95% CI 4.19-7.55), or used one of these promotions (OR 14.05, 95% CI 9.21-21.43). Respondents were more likely to be current smokers if they received a tobacco promotion (OR 2.64, 95% CI 2.02-3.45) or if they used one of these promotions (OR 1.93, 95% CI 1.31-2.85).

**Conclusions:**

Our study suggests that tobacco industry websites reach medical students and young physicians in a middle-income country with their marketing promotions. Current or proposed legislation to ban tobacco advertising needs to include Internet sites and related social media.

## Introduction

Tobacco use constitutes the leading global cause of preventable death with approximately one death every six seconds, accounting for 10% of all adult deaths [1]. There are at least one billion smokers in the world and 80% of them live in low- and middle-income countries [1]. Argentina, a South American middle-income country, has a prevalence of tobacco smoking of 27.1% [2]. Tobacco industry investment in marketing and advertising in Argentina reached approximately US $18 million in 2002. Although Argentina’s president signed the World Health Organization (WHO) Framework Convention on Tobacco Control in 2003, ratification has been delayed so far by the active lobbying of regional legislators from tobacco-growing provinces influenced by tobacco industry positions [3].

The Internet is now one of the most widely used communication channels in the world. Although many countries have restricted advertising of harmful products such as tobacco, Internet-based marketing has the potential to avoid such restrictions [4] while reaching a large number of people. Thus, Internet advertising has become an attractive option for promoting tobacco products and the rise in use of social networking and user-generated content websites is increasing product promotion through electronic media. In a study from 2010, more than 500 Facebook pages were related to British American Tobacco products [5]. Studies that analyzed smoking content on the site YouTube reported the presence of several pro-tobacco videos [6,7], consistent with indirect marketing activity by tobacco companies or their proxies [6]. In Argentina, almost 5 million homes have broadband Internet access covering at least 55% of the population [8,9]. Several cigarette brands have designed websites for the local market that promote user participation whenever possible and target specific products. For example, it is not unusual for a person reached by a marketing promotion to be required to go online in order to participate in a lottery or to obtain a gift, and such promotions usually come included in cigarette packages. Depending on the specific website, content includes online chatting and online gaming, and allows users to provide brand marketing ideas and to participate in marketing campaigns. For example, RJ Reynolds designed a strategy where consumers could comment on the packaging design of four cigarette flavors on the Internet [10].

Health professionals have the potential to play a fundamental role in tobacco control strategies. The WHO has called on health professional organizations to encourage and support their members to be role models by not using tobacco products and by promoting a tobacco-free culture [11]. It has been shown that physicians’ smoking status can impact their interaction with patients and may influence their cessation counseling practices [12-14]. In Argentina, smoking prevalence among physicians to date is similar to that of the general population, unlike what has happened in the United States [15,16]. As new cohorts of physicians enter the medical profession, their smoking behaviors and attitudes will influence tobacco prevention and control efforts. In this context, current medical students and recent graduates constitute an important study population, given their potential impact on the health care system and the population’s health and the fact that these students have grown up with a common exposure to generalized use of new information technologies. Several survey studies around the world have estimated the prevalence of tobacco smoking among medical students and reported rates as low as 2.8% in Uganda and as high as 43.3% in Albania [17]. This variation can also be observed among high-income countries, with a smoking rate of 3.8% among medical students in Wales [18], 25.2% in Berlin, Germany [19], and 20.8% among 350 health professional students in Texas [20]. A survey conducted in 2005 among a sample of third-year medical students in Buenos Aires, Argentina, showed that 35.5% (33.4% of men and 36.5% of women) were current smokers [17]. This prevalence was one of the highest in South America compared to smoking rates reported from Brazil (19.5% of men and 14.6% of women) and Chile (27.1% of men and 29.3% of women) [21].

The relationship between Internet marketing strategies by the tobacco industry and its effect on young adults needs further study. Given that medical students in Argentina smoke at similar rates as the general population and they constitute an especially important target population in implementing national tobacco control strategies, we considered that they would be an appropriate group in which to evaluate the effect of tobacco industry marketing strategies. This study reports on a survey of the use of tobacco industry-sponsored websites among a population of current and recent medical students in Buenos Aires, Argentina.

## Methods

### Setting, Sampling, and Procedures

In 2011, a survey was conducted among current and former medical students in Ciudad de Buenos Aires, Argentina and this analysis focused on the reported use of tobacco industry-sponsored websites. Buenos Aires is the largest city in Argentina and the school of medicine at the University of Buenos Aires (UBA) has over 15,000 students enrolled in the 6-year program [22].

Participants’ email addresses were obtained from the list of students enrolled in the required one-year rotating internship that constitutes the sixth year of medical school, from the Pharmacology Department of the UBA School of Medicine and the largest teaching hospital affiliated with UBA (Hospital de Clínicas Jose de San Martin). This survey was conducted between March and May 2011. The Institutional Review committee of the Hospital de Clínicas, UBA, Argentina, approved the protocol.

Potential participants were invited via email to complete an online, self-administered Spanish-language survey regarding tobacco smoking behaviors and their beliefs and attitudes toward tobacco industry marketing. Each email message provided a unique link to the survey in order to prevent multiple entries from the same email account. Participants initially had to complete a consent form, where they were informed about the study objectives, the length of the instrument, and were reassured about the voluntary and confidential character of the survey. To be eligible, a person had to be between 18 and 30 years old, a former or current medical student at UBA, and had to report on their smoking status. In addition to the initial email invitation to participate, an additional 14 reminder messages were sent over a period of 2-3 months. A lottery for theater tickets was used as an incentive. We attempted to contact all persons on the lists obtained. The survey was conducted using DatStat Illume software. Data was password protected and stored with limited access to research team members only.

### Development of Survey Items

The survey included questions regarding tobacco use, intention to quit, knowledge of tobacco’s health effects, and training in helping patients quit. These items were adapted from the Spanish-language version of the Global Health Professionals Survey (GHPS)*,* a survey of third-year students attending dental, medical, nursing, or pharmacy schools [23], from a questionnaire previously developed for a study of youth in Jujuy, Argentina [24], and from a survey developed by the research team for a cessation study in clinical settings. The smoking media literacy (SML) scale that was developed in the United States and adapted for use in Argentina and is used to assess smoking media literacy and its relationship to smoking history among high school students was included [25,26]. English items underwent forward-backward translation from English to Spanish and were reconciled by three native Spanish-speaking Argentinean staff.

We developed additional items regarding respondents’ access to tobacco brand websites and the use of their marketing promotions. Linguistic and content validation was conducted through a series of semi-structured interviews with medical students. The research team revised the items for a final version of the questionnaire. Pilot testing of the instrument was conducted with resident physicians recently graduated from medical school evaluating situational factors and time of administration. Instructions were developed to address the importance of providing accurate answers and the confidential nature of the survey.

Age, gender, and student status (current medical student or recently graduated physician) were asked. Due to the association with smoking [24,27], screening for depression symptoms was ascertained by asking whether the respondent had felt sad or blue or had felt little interest or pleasure in doing things in the previous month [28,29]. An affirmative answer to either of these questions was considered positive screening for depression.

### Use of Internet and Tobacco Brand Websites

Participants were asked about how frequently they used the Internet, whether they remembered having seen a cigarette advertisement on the Internet, if they have ever received a personally addressed marketing promotion or free tickets to a cultural event from a tobacco company with a specific brand, and if yes, if they ever used it. “Access to a tobacco website” was defined as having visited an institutional or promotional cigarette brand website at least once in their lifetime. Among participants who accessed a tobacco website, we asked if they only accessed them when the cigarette box included a marketing promotion that required accessing a website to obtain a free gift or to participate in a lottery or if they accessed the sites even if they had no intention of participating in the marketing promotion. In each case, we inquired about the frequency of accessing those sites (“from once a day or more” to “once a month or less”).

### Smoking Behavior

Respondents were considered “ever smokers” if they tried at least one cigarette puff in their lifetime and “never smokers” if they had never tried even a puff. Current smokers reported smoking at least one puff in the 30 days prior to the survey. Among current smokers, a “daily smoker” smoked at least one cigarette each day of the last 30, while a “non-daily smoker” (still a current smoker) did not smoke every day. A “former smoker” is a person that had tried a cigarette at least once but had not smoked in the previous 30 days.

### Data Analysis

Descriptive analysis compared the sample by gender and smoking status. Statistical significance was defined with a two-sided alpha of .05. Using multivariate logistic regression, we constructed different models where the outcomes were whether the participant ever accessed a tobacco brand website or was a current smoker. Models were adjusted for age, gender, student status, presence of depression, daily use of Internet, had seen tobacco advertisements on the Internet, received a tobacco marketing promotion addressed to them, and used a tobacco marketing promotion addressed to them. The model where the outcome was “access a tobacco brand website” was also adjusted for “smoking status”. The model where the outcome was “current smoker” was also adjusted for “access a tobacco brand website”. In these models, each variable was adjusted for all the others.

## Results

### Overview

Of 4969 unique names and email addresses included in the databases obtained, 35.08% (1743/4969) completed the survey. Among these 1743 respondents, 84 were excluded from the sample because they had never been in medical school (n=28), were older than 30 years (n=48), or they did not report on their smoking status (n=8). The final sample for analysis included 1659 current or recently graduated medical students. Because some participants did not complete the whole survey and some questions were only shown conditioned on a previous response, the number of answers for a specific question may be less than the total number of participants.

### Demographics and Exposure to Tobacco Marketing

Table 1 summarizes the characteristics of the sample and reported use of Internet outcomes. Among the 1659 participants, 73.06% (1212/1659) were women, 55.70% (924/1659) were current students, 43.58% (723/1659) were physicians, and 0.72% (12/1659) had dropped out of medical school by the time of the survey. Close to one-third of the participants (30.32%, 503/1659) were between 20 and 25 years, 37.25% (618/1659) were between 26 and 27 years, and 32.43% (538/1659) were between 28 and 30 years old. The mean age was 26.6 years (SD 1.9). Of the respondents, 26.88% (446/1659) reported remembering seeing a tobacco advertisement on the Internet but a majority (51.05%, 847/1659) did not recall. A tobacco marketing promotion specifically addressed to the participant was received by 29.05% (482/1659), and 10.07% (167/1659) used at least one of these promotions (Table 1).

Women were more likely to report positive screening for depression (55.12% vs 45.19%; *P*<.001). Men were more likely to report having seen a tobacco advertisement on the Internet (31.77% vs 25.08%; *P*=.001), to have received a tobacco marketing promotion personally addressed to them (31.32% vs 28.22%; *P*=.03), to have used that marketing promotion offer (11.86% vs 9.41%; *P*=.02), and to have accessed a tobacco-sponsored website (22.37% vs 18.23%; *P*=.01).

**Table 1:** Smoking behavior and exposure to tobacco marketing among current and recent medical students (n=1659), Buenos Aires, Argentina, 2011.

Participant characteristics		n (%)
**Age (years)**		
	20-25	503 (30.32)
	26-27	618 (37.25)
	28-30	538 (32.43)
**Gender**		
	Male	447 (26.94)
	Female	1212 (73.06)
**Student status**		
	Currently enrolled student	924 (55.70)
	Graduated as physician	723 (43.58)
	Left medical school without degree	12 (0.72)
**Smoking status**		
	Never smoker	348 (20.98)
	Former smoker	858 (51.72)
	Current smoker	453 (27.31)
**Depressive symptoms**		
	Positive	870 (52.44)
**Internet outcomes** ^a^		
	Has seen tobacco ad on Internet	446 (26.88)
	Received tobacco promotion^b^	482 (29.05)
	Used tobacco promotion^c^	167 (10.07)
	Accessed tobacco brand website	321 (19.35)

^a^“Do not remember” responses were categorized as “No”.

^b^Have received a promotion or invitation from a tobacco brand addressed specifically to the respondent.

^c^Have used a promotion or invitation from a tobacco brand addressed specifically to the respondent.

### Smoking Behavior and Use of Tobacco Brand Websites

Most of the participants reported having smoked at least once (79.02%, 1311/1659) with 27.31% (453/1659) being current smokers. Table 2 presents the characteristics of current smokers and of those participants who ever accessed a tobacco-sponsored website. Among current smokers, 71.3% (323/453) were women and 58.3% (264/453) were current medical students. Most smokers (73.1%; 331/453) reported their first cigarette more than 60 minutes after waking up and 75.3% (341/453) smoked 10 or fewer cigarettes per day. A majority of smokers (73.1%, 331/453) intended to quit within 6 months and 32.9% (149/453) reported smoking in the medical school buildings. Almost half of them reported having received a tobacco promotion or invitation personally addressed to them (49.5%; 224/453) and 21.4% (97/453) used such promotion at least once. Among current smokers, 40.4% (183/453) reported accessing a tobacco brand website.

Almost 20% (321/1659) of the participants reported having accessed a tobacco brand website at least once in their lifetime. Of this group, 68.9% (221/321) were women, 60.4% (194/321) were current medical students, and 57.0% (183/321) were current smokers. Having seen a tobacco ad on the Internet was reported by 44.9% (144/321) of these participants; 68.5% (220/321) received a personally addressed tobacco marketing promotion or invitation while 39.9% (128/321) used one of the marketing promotions (Table 2). Almost all of them accessed these sites only when it was necessary for participating in a marketing promotion or using the promotion to obtain a gift (93.8%, 301/321). Most people logging on for marketing promotions reported entering once a month or less (58.9%, 189/321), while 25.5% (82/321) reported accessing the tobacco industry Internet sites once a week or more. Only 19 participants responded that they accessed the website for reasons other than marketing promotions. When the reason for going to the website was other than a marketing promotion, 73.7% (14/19) used the website once a month or less, while only 15.8% (3/19) used the sites once a week or more.

**Table 2:** Smoking behavior and use of tobacco brand websites among medical students and physicians (n=1659), Buenos Aires, Argentina, 2011.^a^

Participant characteristics		Current smoker, n (%) (n=453)	Accessed tobacco brand website, n (%) (n=321)
**Age (years)**			
	20-25	127 (28.0)	89 (27.7)
	26-27	175 (38.6)	113 (35.2)
	28-30	151 (33.3)	119 (37.1)
**Gender**			
	Male	130 (28.7)	100 (31.2)
	Female	323 (71.3)	221 (68.9)
**Student status**			
	Currently enrolled student	264 (58.3)	194 (60.4)
	Graduated as physician	184 (40.6)	123 (38.3)
	Left medical school without degree	5 (1.1)	4 (1.3)
**Depressive symptoms**			
	Positive	255 (56.3)	187 (58.3)
**Internet outcomes** ^b^			
	Has seen tobacco ad on Internet	121 (26.7)	144 (44.9)
	Received tobacco marketing promotion^c^	224 (49.5)	220 (68.5)
	Used tobacco marketing promotion^d^	97 (21.4)	128 (39.9)

^a^Totals may not equal 100% due to rounding.

^b^“Do not remember” responses were categorized as “No”.

^c^Have received a marketing promotion from a tobacco brand addressed specifically to the respondent.

^d^Have used a marketing promotion from a tobacco brand addressed specifically to the respondent.

### Multivariate Analysis

Unadjusted and adjusted logistic regression models assessed the association of age, gender, smoking status, student status, positive depression screen, daily use of Internet, seen tobacco ad on Internet, received a tobacco marketing promotion addressed to the participant, and used a tobacco marketing promotion addressed to the participant with the outcome of accessing a tobacco industry website. In these models, missing data for specific variables reduced the sample size for each model by no more than 2.7% (final n=1615). In adjusted models, participants were more likely to have accessed a tobacco brand website at least once if they were former or current smokers (OR 2.45, 95% CI 1.42-4.22 and OR 8.12, 95% CI 4.66-14.16 respectively), or if they reported having seen a tobacco advertisement on the Internet (OR 2.44, 95% CI 1.77-3.37), received a tobacco marketing promotion personally addressed to them (OR 5.62, 95% CI 4.19-7.55), or used one of these marketing promotions (OR 14.05, 95% CI 9.21-21.43) (Table 3).

Using similar adjusted models but with current smoking as the outcome, respondents were less likely to be current smokers if they reported having seen a tobacco advertisement on the Internet (OR 0.59, 95% CI 0.45-0.79) and more likely to be a smoker if they received a tobacco marketing promotion personally addressed to them (OR 2.64, 95% CI 2.02-3.45) or if they used one of these marketing promotions (OR 1.93, 95% CI 1.31-2.85).

**Table 3:** Predictors of medical students and physicians accessing tobacco brand websites, Buenos Aires, Argentina, 2011.

			Access a tobacco brand website OR, 95% CI	
Variable		Referent	Unadjusted	Adjusted
Age^a^		Continuous	1.07 (1.001-1.14)^d^	1.10 (1.004-1.20)^d^
**Smoking status** ^a^				
	Current smoker	Never	13.29 (7.97-22.18)^e^	8.12 (4.66-14.16)^e^
	Former smoker		2.97 (1.78-4.97)^e^	2.45 (1.42-4.22)^e^
**Daily use of Internet** ^a^				
	Yes	No	1.43 (0.89-2.29)	1.14 (0.64-2.04)
**Seen tobacco ad on Internet** ^a^				
	Yes	No	2.67 (2.07-3.45)^e^	2.44 (1.77-3.37)^e^
**Received tobacco marketing promotion** ^b^				
	Yes	No	8.62 (6.57-11.31)^e^	5.62 (4.19-7.55)^e^
**Used tobacco marketing promotion** ^c^				
	Yes	No	21.34 (14.46-31.50)^e^	14.05 (9.21-21.43)^e^

^a^These models are adjusted for the following variables: age, gender, smoking status, student status, depression, daily use of Internet, seen tobacco ad on Internet, received a tobacco marketing promotion addressed to them, and use a tobacco promotion addressed to them.

^b^This model is not adjusted for “used tobacco marketing promotion”.

^c^This model is not adjusted for “received tobacco marketing promotion”.

^d^ *P*<.05

^e^ *P*≤.001

## Discussion

### Principal Findings

This is, to our knowledge, the first study that reports on the access and use of websites sponsored by the tobacco industry by medical students and physicians. Although other studies have described tobacco industry marketing promotions on the Web, we did not identify any that asked about the amount of use of these sites in a defined population. In the study population, nearly 20% of the respondents reported having accessed a website sponsored by the tobacco industry at least once, with almost all of them accessing the sites only when it was necessary for using or participating in a marketing promotion. This indicates that online marketing promotions for free gifts or tickets to cultural events are an effective way of delivering tobacco advertising and reaching a young adult population––even medical students and recently graduated physicians. This study also found that participants who received a marketing promotion or an invitation personally addressed to them were more likely to be current smokers and to have accessed one of these websites, even when adjusting for other variables. Intriguingly, respondents who reported having seen a tobacco advertisement on the Internet were less likely to be current smokers. One possible explanation for this observation would be that people who do not smoke are more aware of tobacco advertising than those who do smoke and thus more likely to report seeing it on a survey.

The implications of these findings are that the tobacco industry identifies ways to market to their target population while voluntarily accepting “advertising bans” in print media, radio, and television. Tobacco industry marketing has been regulated over the past decades in most countries, driven in part by the WHO Framework Convention on Tobacco Control endorsing “a comprehensive ban on advertising, marketing promotion, and sponsorship” as a way to reduce tobacco consumption. In fact, implementing a complete advertising ban is one of the evidence-based policies included in modeling full implementation of tobacco control policies in Argentina [30]. However, the Internet remains mostly unregulated for tobacco advertising, marketing promotion, and sponsorship and thus undermines the purpose of those restraints. Our study suggests that tobacco industry online strategies let companies interact directly with consumers, allowing for active user participation, and avoid the advertisement bans being proposed and implemented.

Health professionals possess the greatest potential of any occupation to promote a reduction in tobacco use at the population level [31]. Physicians and other health professionals may contribute to tobacco control by acting as role models, by providing counseling and smoking cessation treatment, and by publically advocating for comprehensive tobacco control public policies [32]. Moreover, physician smoking status may affect their willingness to initiate cessation interventions in their patients and their effectiveness when providing counseling [12-14]. The results of this study indicate that the prevalence of current smoking among medical students and physicians was lower than the one found in the 2005 medical student survey (27.3% vs 35.5%) [17]. This seems to be consistent with the trend toward lower smoking rates in Argentina’s general population, which has shown a reduction over four years based on the national risk factor surveys [2]. It also gets closer to the prevalence found in other South American countries [21]. However, although our sample denominator was large, it was not limited to third-year students only.

### Limitations

Our study has several limitations to consider. First was the relatively low response rate of the sample denominator to conduct the survey. Although this collaboration rate is similar to other studies using Web-based surveys [20,33,34], generalizations based on these data need to be cautious. Unfortunately, we do not have information about the demographics of non-respondents in order to compare their characteristics with the participants who actually completed the survey. However, the study population was mostly women and this reflects the composition of the UBA medical school where women account for two-thirds of the student body [22]. The proportion by age groups is also what would be expected based on UBA enrollment. Another limitation is the fact that we did not ask how many times a person accessed one of these websites (only once or more) and when they actually accessed the sites. Thus, we cannot be certain that the students were current smokers at the time of website access. Finally, use of cross-sectional data prevents establishing any causal inference or temporal relationships from our results.

### Conclusions

Despite these limitations, our study suggests that tobacco industry websites do reach young adult medical students and physicians in a middle-income country with their marketing promotions. This strategy may be essential in maintaining current smoking status in this group and young adults in general. Internet advertisement may also give the tobacco industry an opportunity to improve their public image while undermining the dissemination of accurate public health information. In June 2011, Argentina’s Congress approved a law that regulates tobacco products [35]. The text of the law bans publicity, marketing promotions, and sponsorship of any tobacco product, direct or indirect, through any media. Although the law has now been implemented by the Executive Branch, our study indicates the need for further research to analyze the impact of this law on tobacco-sponsored websites and social media communication. Legislation to ban tobacco advertising and marketing promotions needs to explicitly include Internet sites and related social media.

## Abbreviations

GHPS: Global Health Professionals Survey
UBA: University of Buenos Aires
WHO: World Health Organization
